# Early inner retinal thinning and cardiovascular autonomic dysfunction in type 2 diabetes

**DOI:** 10.1371/journal.pone.0174377

**Published:** 2017-03-23

**Authors:** Jin A. Choi, Hyo Won Kim, Jin-Woo Kwon, Yun-sub Shim, Dong Hyun Jee, Jae-Seung Yun, Yu-Bae Ahn, Chan Kee Park, Seung-Hyun Ko

**Affiliations:** 1 Department of Ophthalmology and Visual Science St. Vincent’s Hospital, College of Medicine, The Catholic University of Korea, Seoul, Korea; 2 Division of Endocrinology and Metabolism, Department of Internal Medicine, St. Vincent’s Hospital, College of Medicine, The Catholic University of Korea, Seoul, Korea; 3 Department of Ophthalmology and Visual Science Seoul St. Mary’s Hospital, College of Medicine, The Catholic University of Korea, Seoul, Korea; University of Michigan, UNITED STATES

## Abstract

**Background:**

To investigate changes in the neural retina according to the presence of retinal nerve fiber layer (RNFL) defects in type 2 diabetes, and to determine the association between inner retina thickness and the severity of diabetic complications.

**Methods:**

We studied non-glaucomatous patients with type 2 diabetes and control subjects Circumpapillary RNFL and macula ganglion cell-inner plexiform layer (GCIPL) thicknesses were measured by spectral-domain optical coherence tomography. In patients with type 2 diabetes, a cardiovascular autonomic function test (AFT) was performed, which included the heart rate parameter of beat-beat variation—with deep breathing, in response to the Valsalva maneuver, and on postural change from lying to standing. The results of each test were scored as 0 for normal and 1 for abnormal. A total AFT score of 1 was defined as early cardiovascular autonomic neuropathy (CAN), and an AFT score≥ 2 as definite CAN.

**Results:**

We compared control eyes (n = 70), diabetic eyes with RNFL defects (n = 47), and eyes without RNFL defects (n = 30). The average RNFL and GCIPL thicknesses were significantly different among groups (all, *P*<0.05). On post-hoc testing, diabetic eyes with RNFL defects had a significantly thinner average GCIPL thickness than those without RNFL defects. On multivariate analyses, significantly thinner average GCIPL was seen in early CAN staging (B = -4.32, *P* = 0.016) and in definite CAN staging (B = -10.33, *P*<0.001), compared with no CAN involvement, after adjusting for confounding parameters.

**Conclusions:**

Cardiovascular autonomic dysfunction was associated with early neurodegenerative changes in type 2 diabetes.

## Introduction

It is widely recognized that the early neurodegenerative changes that characterize diabetes involve dysfunction and degeneration of retinal neurons even before the manifestation of vascular symptoms.[1, 2] High blood glucose induces apoptosis in retinal neural cells.[3] Increased neurofilament phosphorylation,[4] glial cell reactivity during metabolic stress,[5, 6] microglial activation, and altered glutamate regulation [7] are also involved in neurodegeneration in the diabetic retina. These early changes especially involve the inner retina, as shown by the reduction in thickness of the retinal nerve fiber layer (RNFL)[8] and loss of ganglion cell bodies.[9]

Optical coherence tomography (OCT) can quantitatively measure the thickness of the retina. Recent advances in OCT technology enabled us to measure the inner layer of the macula, consisting of the ganglion cell layer and inner plexiform layer (GCIPL) using a ganglion cell analysis (GCA) algorithm. In diabetes, early thinning of the macular GCIPL thickness has been reported even before visible vascular signs of diabetic retinopathy (DR).[10] Neurodegeneration in the early stages of diabetes compromises the functions of neurons, resulting in subtle but significant impairment of vision.[11] Despite its clinical importance, little is known about the systemic risk factors associated with inner retina thinning in diabetes.

Thinning of the inner retina correlates with the distribution of RNFL visibility in fundus examinations.[12] Reduction in RNFL visibility, described as an RNFL defect, does not develop in normal eyes. Therefore, the presence of an RNFL defect indicates a pathological change, and is a highly specific marker for optic nerve head abnormalities.[13, 14] The presence of RNFL defects has been associated with several conditions such as arterial hypertension [15], small vessel disease, [16] cerebrovascular infarction,[17] and glaucoma.[15] In type 2 diabetes patients, the RNFL defects that develop in the early stages of retinal changes have been associated with increased urinary albumin excretion.[18] Also, the location of RNFL defects during diabetes is associated with diabetic peripheral neuropathy and cardiovascular autonomic neuropathy (CAN).[19]

Despite the clinical significance of RNFL defects as a diagnostic tool for optic nerve abnormalities, the detection of RNFL defects using red-free fundus photography is sometimes limited; it is a qualitative measurement, and the results are dependent on the operator.[20, 21] It is therefore important to determine the association of quantitative OCT parameters with the severity of diabetic complications.

In this study, we examined the differences in the circumpapillary RNFL and macular GCIPL thicknesses according to the presence of RNFL defects in patients with type 2 diabetes, and then investigated the systemic potential risk factors affecting macular GCIPL thickness.

## Methods

### Study population

This study included consecutively enrolled patients with type 2 diabetes and age- and sex-matched control subjects (40–80 years of age) who underwent ophthalmic examinations and spectral domain OCT from July 2014 to June 2015 at St. Vincent’s Hospital, Suwon, South Korea. This retrospective chart review was approved by the Institutional Review and Ethics Boards of the Catholic University, St. Vincent’s Hospital (local Institutional Review Board number: VC14RISI0153). The institutional review board waived the need for a written consent from the participants, because of the retrospective design. Patient information was anonymized and de-identified prior to analysis. The study design followed the tenets of the Declaration of Helsinki for biomedical research.

Subjects with type 2 diabetes were included if they had no signs of a glaucomatous optic disc (focal or generalized narrowing, disappearance of the neuroretinal rim, disc hemorrhage, or cup-to-disc asymmetry > 0.2). They were required to have normal visual field (VF) results during the follow-up. A normal VF examination was defined as a glaucoma hemifield test result within normal limits, and total and pattern standard deviation values associated with probabilities of normality > 5%. Patients with type 1 diabetes, proliferative DR, neovascular glaucoma, macular edema, or a known past history of macula edema were excluded from the study. Patients who underwent interventions such as panretinal photocoagulation or intravitreal injection, and those who were using anti-glaucoma eye drops, were also excluded. Patients with acute complications of diabetes at present, or with arrhythmia, or with severe illnesses such as heart failure, liver cirrhosis, alcoholism, severe infection, or malignancy, were excluded. Patients with type 1 diabetes or gestational diabetes, chronic kidney disease stage 3 or higher, and end-stage renal disease were also excluded.

The control group included subjects with an intraocular pressure < 21 mmHg, a normal optic disc appearance upon examination of color stereoscopic photographs (an intact neuroretinal rim without peripapillary hemorrhage, thinning, or localized pallor), the absence of any RNFL abnormality visible on red-free fundus photographs, normal VF test results, and no systemic disease such as diabetes or hypertension. All subjects were required to have a best-corrected visual acuity (BCVA) of 20/40 or better, a spherical equivalent within ± 5.0 diopters, and open angles on gonioscopy.

### Ophthalmic examination

All subjects underwent a comprehensive ophthalmic examination, including a review of medical and ocular histories, measurement of BCVA, Goldmann applanation tonometry, slit-lamp biomicroscopy, gonioscopic examination, dilated funduscopic examination, stereoscopic optic disc photography, red-free RNFL photography (CF-60UD; Canon, Tokyo, Japan), standard automated perimetry measurements using the 24–2 SITA program (Humphrey Visual Field Analyzer; Carl Zeiss Meditec, Dublin, CA, USA), and OCT (Cirrus OCT; Carl Zeiss Meditec, CA, USA). DR was graded by retinal specialists (DHJ and JWK), based on the International Clinical Diabetic Retinopathy Severity Scale.[22]

To determine RNFL defects using fundus photography, two experienced ophthalmologists (CJA and JWK), who were blinded to the subject’s identity, evaluated the red-free photographs to detect any RNFL defects. An RNFL defect was defined as described in definitions of previous studies.[2, 23] Any cases involving disagreement of the presence of RNFL defects between the two observers were excluded from the study.

### Systemic evaluation

Diabetes was diagnosed in subjects with a fasting plasma glucose ≥ 126 mg/dL or symptoms of diabetes and a casual plasma glucose concentration of ≥ 200 mg/dL using an automated enzymatic method.[24] Glycated hemoglobin (HbA1c) levels were measured using high-performance liquid chromatography (Bio-Rad, Montreal, Quebec, Canada) every 3 months. The average HbA1c level during the most recent 12 months (the mean HbA1c) was used in this study. The treatment of diabetes was categorized according to use an oral agent, insulin, or lifestyle modifications alone. Hypertension was defined as systolic blood pressure ≥ 140 mmHg, diastolic blood pressure ≥ 90 mmHg, or the use of hypertensive medications. Serum lipid concentrations of total cholesterol, high-density lipoprotein (HDL) cholesterol, low-density lipoprotein (LDL) cholesterol, and triglycerides were measured enzymatically using an automatic analyzer (Model 736–40; Hitachi, Tokyo, Japan). Smoking status was defined as either current or past smoker. The estimated glomerular filtration rate was calculated using the Modification of Diet in Renal Disease Study equation.[25] From the first-voided spot urine samples, the albumin-to-creatinine ratio (ACR) was calculated.

### CAN assessment

The cardiovascular autonomic function test (AFT) was performed using Ewing’s method. This includes a test for heart rate variability, such as the beat-beat variation with deep breathing, on postural change (from lying to standing), and in response to the Valsalva maneuver, as described previously.[26, 27] Each of the three ratios described above was scored as 0 (*normal*) or 1 (*abnormal*), for a total maximum score of 3. The CAN stage was defined as follows: 0, normal autonomic function; 1, early CAN; and ≥ 2, definite diagnosis of CAN.[28]

### OCT measurements

All participants were imaged by spectral-domain OCT, which comprised an optic-disc scan (optic disc cube, 200 × 200 protocol) and a macular scan (macular cube, 514 × 128 protocol). The circumpapillary scan allowed measurement of RNFL thickness, whereas the macular scan determined the macular GCIPL thickness using the GCA algorithm.[29, 30] The average RNFL thickness and RNFL thickness for each quadrant sector were determined for all participants. The GCA provides the average GCIPL thickness and that for each of the six individual sectors [superior (S), superonasal (SN), inferonasal (IN), inferior (I), inferotemporal (IT), and superotemporal (ST)], in addition to the deviation map, on which areas appear as yellow or red to represent GCIPL thickness less than the lower 5% or 1% of normative data, respectively. In this study, the average and by-sector GCIPL thicknesses were recorded. The average values of S, SN, and ST were designated as superior hemifield GCIPL thicknesses, and the average values of I, IN, and IT were the inferior hemifield GCIPL thicknesses. A sector map with one or more yellow/red colored sectors was considered abnormal. The abnormal deviation map was defined as the presence of yellow/red pixels in the map, as previously described.[31] Only well-focused signal strengths ≥ 7/10 were used.

### Statistical analyses

The reproducibility of interobserver measurements (by CJA and JWK) for the detection of photographic RNFL defects was assessed by calculation of intraclass correlation coefficients (ICCs). Student’s *t*-test and the chi-squared test were used to compare between group means and percentages derived from independent samples. Differences between three groups were determined by analysis of variance (ANOVA) followed by Bonferroni post-hoc testing. For factors that were markedly skewed, log-transformed values were used in the analyses. Crude associations between potential systemic risk factors and macula GCIPL thickness parameters were determined using univariate linear regression analyses. A dummy variable linear regression model was used to investigate the effect of the three CAN gradings on the average GCIPL thickness. To determine the association between the severity of CAN and the average GCIPL thickness, multiple linear regression analyses were used. The dependent variable was average GCIPL thickness. For multiple linear regression analyses, variables with *P* < 0.15 (age, CAN stage, spherical equivalent, total cholesterol, triglyceride level, and the duration of diabetes) in univariate analyses were included in Model 1. In Model 2, potential confounders that could affect the association between CAN stage and the average GCIPL thickness (presence of DR, average HbA1c, smoking, use of statins, antiplatelet activity, and angiotensin-converting enzyme inhibitors or angiotensin receptor blockers) were additionally included. A backward elimination process was used to develop the final multivariate model. Statistical analyses were performed using SPSS for Windows software (ver. 18.0; SPSS Inc., Chicago, IL, USA). A value of *P* < 0.05 was considered statistically significant.

## Results

A total of 333 patients with diabetes underwent ophthalmic examinations between July 2014 and July 2015. Of these, 199 with glaucomatous optic discs or glaucomatous VF losses were excluded, and 14 were excluded because of ambiguous RNFL defects. Six eyes about which the two observers were in disagreement in terms of RNFL defect measurements were also excluded. Also, 37 eyes were excluded because AFT results were lacking. Finally, 77 patients with type 2 diabetes and 70 control subjects were included. The interobserver ICC (95% confidence interval) for the detection of photographic RNFL defects was 0.918 (0.874–0.947).

The mean patient age and diabetes duration were 55.7 ± 10.7 years and 9.3 ± 6.8 years, respectively; 47 subjects (61.0%) had RNFL defects and 30 subjects (39.0%) did not. In total, 51 patients (66.2%) had no DR, 17 patients (22.1%) had mild non-proliferative diabetic retinopathy (NPDR), 8 patients (10.4%) had moderate NPDR, and 1 patient (1.3%) had severe NPDR. The mean age, sex, and spherical equivalent were not significantly different among controls, diabetic patients with RNFL defects, and those without RNFL defects (Table 1). When comparing patients with RNFL defects with those without RNFL defects, abnormal CAN staging was significantly more frequent in patients with RNFL defects (*P* = 0.037), and the log-transformed ACR was higher in subjects with RNFL defects (*P* = 0.045).

**Table 1:** Comparison of characteristics of control subjects and patients according to the presence of retinal nerve fiber layer defect.

	Controls	DM without	DM with	
		RNFL defects	RNFL defects	
n	70	47	30	
Mean age, y	54.5 ± 8.8	56.1 ± 9.1	55.5 ± 11.7	0.735*
Male: female	43:27	23:24	15:15	0.338*
Spherical equivalent, Diopter	-0.74 ± 1.63	-0.51 ± 1.54	-0.25 ± 1.17	0.261*
Mean duration of DM, y		8.4 ± 6.8	10.8 ± 6.9	0.125^†^
Presence of hypertension, n (%)		19 (40.4)	15 (50)	0.483^‡^
Presence of DR, n (%)		12 (25.5)	14 (46.7)	0.083^‡^
Diabetes treatment, n (%)				0.542^‡^
Life style modification		7 (14.9)	2 (6.7)	
Oral agent only		25 (53.2)	18 (60.0)	
Insulin		15 (31.9)	10 (33.3)	
Staging of CAN, n (%)				0.019^‡^
Normal		23 (54.7)	8 (26.7)	
Early		16 (38.1)	14 (46.7)	
Definite		3 (7.1)	8 (26.7)	
Hemoglobin A1c, %		7.8 ± 1.1	8.0 ± 2.0	0.589^†^
eGFR (mml/min per 1.73 mm^2^)		102.1 ± 23.8	101.2 ± 21.3	0.866^†^
ACR (ug/mg creatinine)^§^		1.1 ± 0.6	1.4 ± 0.6	0.045^†^
Total cholesterol (mg/dl)		168.7 ± 31.1	167.9 ± 35.6	0.926^†^
HDL cholesterol (mg/dl)		41.9 ± 8.8	45.0 ± 9.3	0.153^†^
LDL cholesterol (mg/dl)		93.7 ± 27.2	71.7 ± 28.4	0.769^†^
Triglyceride (mg/dl)		169.0 ± 118.1	130.7 ± 61.8	0.074^†^

*RNFL* retinal nerve fiber layer, *DM* diabetes mellitus, *DR* diabetic retinopathy, *CAN* cardiovascular autonomic neuropathy, *eGFR* estimated glomerular filtration rate, *ACR* urinary albumin-to-creatinine ratio, *HDL* high-density lipoprotein, *LDL* low-density lipoprotein.

*ANOVA

^†^independent *t*-test

^‡^chi-square test

^§^Log-transformed variable.

**Fig 1:**
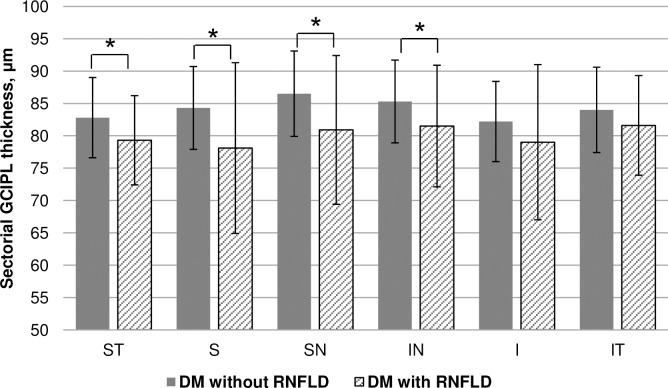
Comparisons of sectorial GCIPL thickness according to the presence of RNFL defect in diabetes. *GCIPL* ganglion cell-inner plexiform layer, *RNFLD* retinal nerve fiber layer defect, *SN* superonasal, *S* superior, *ST* superotemporal, *IN* inferonasal, *I* inferior, *IT* inferoteporal. *independent *t*-test.

Regarding comparison of circumpapillary RNFL parameters, the average RNFL thickness and superior quadrant and inferior quadrant RNFL thicknesses were significantly different among groups (*P* = 0.027, 0.020, and < 0.001, respectively; Table 2) Post-hoc analyses showed no significant difference in the circumpapillary RNFL thicknesses between diabetic patients without RNFL defects and those with RNFL defects. Regarding comparison of the macula GCIPL thickness, the average GCIPL thickness and the superior and inferior hemifield GCIPL thicknesses were significantly different among groups (*P* = 0.001, < 0.001, and 0.016, respectively). Post-hoc analyses showed that diabetic patients with RNFL defects had significantly thinner average GCIPL and superior hemifield GCIPL thicknesses, compared with diabetic patients without RNFL defects. Disc areas were 1.94 ± 0.27 mm^2^ for the control group, 2.02 ± 0.32 mm^2^ for diabetic patients without RNFL defects, and 1.90 ± 0.28 mm^2^ for diabetic patients with RNFL defects (*P* = 0.148). Fig 1 shows the differences in sectorial GCIPL thicknesses according to the presence of RNFL defects in diabetic patients. The ST, S, SN, and IN sectors were significantly thinner in patients with RNFL defects compared with those without RNFL defects (*P* = 0.032, 0.011, 0.011, and 0.046, respectively).

**Table 2:** Comparisons of circumpapillaryRNFL and macula GCIPL thickness parameters among controls, diabetic patients with RNFL defect, and those without RNFL defect.

	Control	DM without RNFL defect	DM with RNFL defect	P value*	Post Hoc test^†^
	Group A	Group B	Group C		
RNFL parameters					
Average RNFL thickness, um	98.3 ± 7.9	94.6 ± 9.5	93.9 ± 10.3	0.027	A = B = C
Superior quadrant	125.7 ± 15.5	121.1 ± 14.9	116.0 ± 18.2	0.020	A>C
Inferior quadrant	127.9 ± 11.8	118.5 ± 14.7	118.2 ± 15.6	<0.001	A>B
Temporal quadrant	72.2 ± 8.4	71.4 ± 11.8	71.3 ± 8.2	0.876	
Nasal quadrant	67.2 ± 7.9	67.3 ± 9.5	70.4 ± 11.0	0.260	
GCIPL parameters					
Average GCIPL thickness	85.6 ± 4.7	84.1 ± 6.0	80.1 ± 9.2	0.001	A>C,B>C
Superior hemifield (SN,S,ST)	86.3 ± 4.9	84.5 ± 6.2	79.5 ± 10.3	<0.001	A>C,B>C
Inferior hemifield (IN,I,IT)	84.9 ± 4.7	83.8 ± 6.1	80.7 ± 9.5	0.016	A>C

*GCIPL* ganglion cell-inner plexiform layer, *RNFL* retinal nerve fiber layer, *SN* superonasal, *S* superior, *ST* superotemporal, *IN* inferonasal, *I* inferior, *IT* inferotemporal.

*ANOVA

^†^Bonferroni test.

Table 3 shows the difference in circumpapillary RNFL and macular GCIPL thicknesses in diabetic patients according to CAN stage. The average circumpapillary RNFL thickness, and the values for the four individual quadrants did not differ significantly among the groups. However, the average, superior hemifield, and inferior hemifield GCIPL thicknesses were significantly thinner with increasing CAN stage (*P* = 0.003, 0.006, and 0.007, respectively).

**Table 3:** Comparisons of circumpapillaryRNFL and macula GCIPL thickness parameters based on the stage of cardiac autonomic neuropathy in subjects with type 2 diabetes.

	Subjects without CAN involvement	Subjects with early CAN	Subjects with definite CAN	P value*	Post Hoc test^†^
n	30	26	10		
RNFL parameters					
Average RNFL thickness, um	96.9 ± 9.1	92.7 ± 9.8	94.0 ± 11.5	0.252	
Superior quadrant	122.3 ± 14.0	115.8 ± 3.5	119.6 ± 14.3	0.309	
Inferior quadrant	121.0 ± 11.9	117.6 ± 14.3	118.3 ± 23.6	0.300	
Temporal quadrant	73.0 ± 12.7	69.0 ± 8.7	72.8 ± 7.3	0.676	
Nasal quadrant	71.8 ± 9.1	67.2 ± 10.6	68.5 ± 8.5	0.174	
Macular GCIPL parameters					
Average GCIPL thickness	86.0 ± 4.9	80.8 ± 6.1	77.9 ± 13.4	0.003	N>E, N>D
Superior hemifield (SN,S,ST)	86.1 ± 5.3	80.5 ± 1.7	77.8 ± 12.3	0.006	N>E, N>D
Inferior hemifield (IN,I,IT)	85.8 ± 4.6	81.4 ± 5.7	77.8 ± 14.5	0.007	N>D

*GCIPL* ganglion cell-inner plexiform layer, *CAN* cardiovascular autonomic neuropathy, *SN* superonasal, *S* superior, *ST* superotemporal, *IN* inferonasal, *I* inferior, *IT* inferoteporal. *N* normal CAN, *E* early CAN, *D* definite CAN.

*ANOVA

^†^Bonferroni test.

Dummy variable regression analyses showed a significantly thinner average GCIPL in early CAN (B = -4.38, *P* = 0.021) and definite CAN (B = -7.33, *P* = 0.006) compared with the absence of CAN involvement (Table 4). Using crude univariate linear regression analyses, other systemic risk factors associated with the average GCIPL thickness were age, total cholesterol, and triglyceride level (*P* = 0.040, 0.044, and < 0.001, respectively; Table 3).

**Table 4:** Univariate associations between systemic risk factors and GCIPL parameters.

	Average GCIPL thickness		Superior hemifield GCIPL thickness		Inferior hemifield GCIPL thickness	
	Regression coefficient(95% Confidence interval)	P	Regression coefficient(95% Confidence interval)	P	Regression coefficient(95% Confidence interval)	P
Age, per 1 yr	-0.17 (-0.33, -0.01)	0.040	-0.18 (-0.36, -0.00)	0.049	-0.16 (-0.32, 0.01)	0.069
Sex	-2.04 (-5.63, 1.55)	0.261	-1.06 (-5.02, 2.90)	0.595	-3.06 (-6.65, 0.53)	0.094
Spherical equivalent, per 1 diopter	-1.20 (-2.51, 0.11)	0.073	-0.81 (-2.27, 0.65)	0.273	-1.60 (-2.88, -0.27)	0.019
SBP, per 1 mmHg	0.09 (-0.07, 0.25)	0.278	0.16 (-0.02, 0.33)	0.080	0.02 (-0.14, 0.18)	0.835
DBP, per 1 mmHg	0.06 (-0.16, 0.28)	0.566	0.13 (-0.11, 0.37)	0.284	-0.01 (-0.23, 0.22)	0.966
eGFR per 1 mml/min per 1.73 mm^2^	0.04 (-0.04, 0.12)	0.285	0.04 (-0.05, 0.12)	0.370	0.04 (-0.40, 0.12)	0.332
ACR, per1 ug/mg creatinine*	-0.65 (-3.54, 2.26)	0.658	-1.22 (-4.40, 1.95)	0.444	-0.21 (-3.14, 2.72)	0.888
CAN staging	-6.28 (-11.27, -1,30)	0.014	-5.71 (-11.26, -0.16)	0.044	-6.95 (-11.96, -1.94)	0.007
early CAN	-4.38 (-8.08, -0.68)	0.021	-5.03 (-9.09, -0.97)	0.016	-3.46 (-7.26, 0.34)	0.074
definite CAN	-7.33 (-12.45, -2.01)	0.006	-7.68 (-13.30, -2.06)	0.008	-7.13 (-12.39, -1.87)	0.009
Total cholesterol, per 1 mg/dl	0.06 (0.00, 0.11)	0.044	0.07 (0.01, 0.13)	0.032	0.05 (-0.11, 0.10)	0.115
HDL cholesterol, per 1 mg/dl	0.01 (-0.20, 0.21)	0.936	-0.01 (-0.24, 0.21)	0.899	0.03 (-0.18, 0.23)	0.813
LDL cholesterol, per 1 mg/dl	0.03 (-0.40, 0.10)	0.404	0.05 (-0.03, 0.12)	0.225	0.01 (-0.06, 0.08)	0.755
Triglycerides, per 1 mg/dl	0.03 (0.01, 0.05)	<0.001	0.03 (0.02, 0.05)	<0.001	0.03 (0.01, 0.04)	0.003
Duration of diabetes, per 1 yr	-0.22 (-0.48, 0.04)	0.092	-0.26 (-0.54, 0.02)	0.073	-0.20 (-0.45, 0.07)	0.155
Mean hemoglobin A1c, per 1%	-0.74 (-2.57, 1.09)	0.425	-1.26 (-3.25, 0.74)	0.213	-0.24 (-2.09, 1.62)	0.801
Use of statin medication, yes vs. no	1.86 (-1.80, 5.51)	0.314	1.93 (-2.07, 5.94)	0.339	1.76 (-1.94, 5.47)	0.346
DR, yes vs. no	1.00 (-2.82, 4.71)	0.605	1.14 (-3.04, 5.32)	0.588	0.504 (-3.37, 4.38)	0.796

*GCIPL* ganglion cell-inner plexiform layer, *SBP* systolic blood pressure, *DBP* diastolic blood pressure, *eGFR* estimated glomerular filtrationrate, *ACR* urinary albumin-to-creatinine ratio, *CAN* cardiovascular autonomic neuropathy, *HDL* high-density lipoprotein, *LDL* low-density lipoprotein, *RNFL* retinal nerve fiber layer, *DR* diabetic retinopathy.

*Log-transformed variable.

In multivariate Model 1, the presence of early CAN (B = -2.32, *P* = 0.075) and definite CAN (B = -8.65, *P* = 0.001; Table 5) contributed significantly more to the decreased average GCIPL thickness relative to the absence of CAN involvement. Serum triglyceride was also significantly associated with higher average GCIPL thickness (B = 0.027, *P* = 0.001). In multivariate Model 2, the presence of early CAN (B = -4.32, *P* = 0.016) and definite CAN (B = -10.33, *P* < 0.001) also contributed significantly more to the decreased average GCIPL thickness. Serum triglyceride and use of statins were significantly associated with a higher average GCIPL thickness (B = 0.028, *P* = 0.001 and B = 4.36, *P* = 0.008, respectively).

**Table 5:** Multivariate models: Predictors of the average GCIPL thickness on regression analysis

	Model 1		Model 2	
	Regression coefficient	*P* value	Regression coefficient	*P* value
CAN staging, normal-borderline vs. definite-severe				
early CAN	-3.23 (-6.79, 0.34)	0.075	-4.32 (-7.81, -0.83)	0.016
definite CAN	-8.65 (-13.56, -3.74)	0.001	-10.33 (-15.16, -5.55)	<0.001
Triglyceride	0.03 (0.01, 0.04)	0.001	0.03 (0.01, 0.04)	0.001
Statin			4.36 (1.16, 7.57)	0.008

*CAN* cardiovascular autonomic neuropathy, *GCIPL* ganglion cell-inner plexiform layer. For multiple linear regression models between CAN staging and the average GCIPL thickness, parameters were included as follows: model1, age, spherical equivalent, total cholesterol, triglyceride, duration of diabetes (parameters showing *P* < 0.150 in univariate associations), model 2, model 1 plus presence of diabetic retinopathy, mean hemoglobin A1c, smoking, use of statin, antiplatelet, and angiotensin converting enzyme inhibitors or angiotensin receptor blockers. A backward elimination process was used to develop the final multivariate model.

In the GCA deviation map, 70.0% of patients with definite CAN showed abnormal findings, whereas 54.2% of patients with early CAN–and 26.7% patients without CAN–were abnormal (chi-squared test, *P* = 0.025). In the GCA sector map, 30.0% of patients with definite CAN had abnormal findings, whereas no patients with early CAN, and 16.7% of patients with normal CAN, were abnormal (chi-squared test, *P* = 0.016).

## Discussion

**Fig 2:**
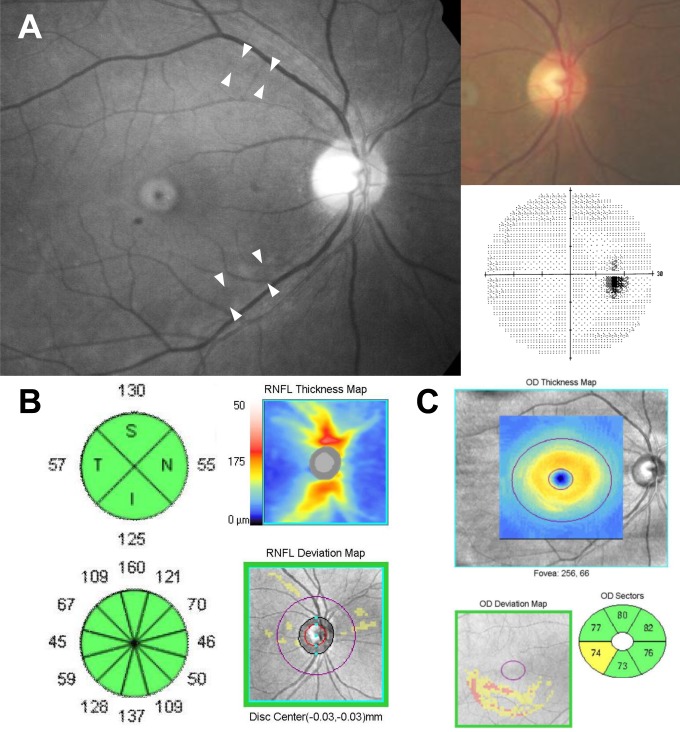
Representative case showing the association between photographic RNFL defect and macular GCIPL thickness. A 57-year old female with a 17-year history of type 2 diabetes exhibited a definite involvement of cardiac autonomic neuropathy. In the red-free photograph, retinal nerve fiber layer (RNFL) defects are observed (A, white arrow). In the quadrant and clock-hour based map, the circumpapillary RNFL thickness is within normal limit (B). However, in the GCA deviation map and sector map exhibit abnormal finding (C)

In the present study, diabetic patients with RNFL defects showed a lower macular GCIPL thickness compared with those without RNFL defects. The average macular GCIPL thickness was significantly decreased with the severity of CAN after adjusting for potential confounding factors. Collectively, our results suggest that diabetic cardiovascular autonomic neuropathy is associated with the early neurodegenerative changes of type 2 diabetes, as shown in representative case (Table 5; Fig 2).

Diabetic cardiovascular autonomic neuropathy is one of the most common and serious complications of diabetes.[26, 32, 33] Damage to the autonomic nerve fibers that innervate the heart and blood vessels causes abnormalities in heart rate control and vascular dynamics. [26] In patients with diabetes, the extent of CAN is inversely related to survival and quality-of-life,[26, 34] and is associated with cardiovascular events, stroke, and other diabetic complications such as severe hypoglycemia and chronic kidney disease.[27, 28, 35] CAN may be detected at the time of diabetes diagnosis; poor glycemic control plays a central role in the development and progression of CAN. [26] In the present study, we used Ewing’s method to determine the extent of CAN. [36] In contrast to other diabetic, autonomic neuropathic complications, which are usually diagnosed by exclusion because of the absence of confirmatory diagnostic tools, CAN can be evaluated both easily and noninvasively on an outpatient basis.

In this study, patients with an RNFL defect showed thinner macula GCIPLs, compared with those without RNFL defects. To the best of our knowledge, this is the first study to report the characteristics of OCT parameters on photographs of RNFL defects in type 2 diabetes. Notably, the RNFL defects originating from the optic nerve head accompanied significant changes in the macula area. RNFL defects are one of the early signs of neurodegenerative changes in diabetes.[2, 13] However, RNFL defects also reflect systemic conditions such as arterial hypertension,[15] stroke,[17] and small vessel disease. [16]. In patients with normal tension glaucoma, those with central VF defects on initial presentation have higher levels of systemic vascular risk factors and disc hemorrhage.[37, 38] Based on our present results, the early neurodegenerative changes represented by RNFL defects may accompany generalized changes in the ganglion cells of the macula.

Although RNFL defects on red-free fundus photographs provide useful clinical information, detection of RNFL defects using these photographs represents a qualitative measurement, so the results could be subjective and dependent on the operator.[20, 21] The macular GCIPL topography is less variable among normal individuals compared with that of other diagnostically important structures, such as the optic disc and RNFL.[39] In our study, subjects with definite CAN showed significantly more abnormal findings in the GCA deviation and sector maps (chi-squared test, *P* = 0.025 and 0.016, respectively). Corneal neurodegenerative changes detected by corneal confocal microscopy have also been considered as early biomarkers of diabetic polyneuropathy.[40] In this regard, it is therefore possible that the macular GCIPL thickness represents a useful parameter for the early detection of neurodegenerative changes in diabetic patients.

In regional analyses, the GCIPLs, especially at the superior sectors (ST, S, and SN), were significantly thinner in patients with RNFL defects (Fig 1). As previously reported, RNFL loss in diabetic patients usually occurs in the superior hemisphere. Our observation that the superior GCIPL thickness was thinner in diabetic patients with RNFL defects is consistent with these findings.

Crude univariate associations of systemic factors with macula GCIPL thickness, and higher serum triglycerides were also significantly associated with higher average GCIPL, and superior and inferior hemifield GCIPL, thicknesses (*P* <0.001, <0.001, and 0.003, respectively). Consistent with our data, Sasaki et al. [41] reported that a higher LDL cholesterol level was associated with increased macular thickness in diabetic patients without macula edema. Lipid-lowering agents such as statins decrease vascular disease and may increase the lifespan.[42] However, the use of statin medication was not significantly associated with the average GCIPL thickness on univariate analyses in our study. Further prospective studies are therefore needed to investigate possible associations of the overall serum lipid status with the retinal structures.

This study had some limitations. First, we did not include patients who did not receive complete ophthalmic examinations, spectral domain OCT, and systemic evaluations, which may have affected the interpretation of the results, including due to selection bias). Second, we excluded eyes with proliferative DR and macular edema. Furthermore, most patients (64.9%) had no DR. However, the presence of DR may result in potential errors in OCT measurements, such as misidentification of the outer layer and off-center artifacts.[43] Finally, causal relationships cannot be inferred, such that the results of our cross-sectional study should be interpreted with caution.

In summary, the results of our study emphasize the potential utility of macula GCIPL parameters as markers of early neurodegenerative changes during diabetes. Careful clinical attention to diabetic patients with abnormal thinning in macular GCIPL is recommended, when considering that the macular GCIPL thickness significantly decreases with the severity of CAN. Further prospective studies are needed to confirm the longitudinal changes in macular OCT parameters associated with the severity of systemic complications during diabetes.
